# Production of therapeutic proteins in the chloroplast of *Chlamydomonas reinhardtii*

**DOI:** 10.1186/s13568-014-0057-4

**Published:** 2014-08-13

**Authors:** Alma Lorena Almaraz-Delgado, José Flores-Uribe, Víctor Hugo Pérez-España, Edgar Salgado-Manjarrez, Jesús Agustín Badillo-Corona

**Affiliations:** 1 Unidad Profesional Interdisciplinaria de Biotecnología, Instituto Politécnico Nacional, Av. Acueducto SN Col. Barrio la Laguna Ticoman, Mexico City, 07340, México

**Keywords:** Chlamydomonas, Chloroplast transformation, Recombinant therapeutic proteins

## Abstract

Chloroplast transformation in the photosynthetic alga *Chlamydomonas reinhardtii* has been used to explore the potential to use it as an inexpensive and easily scalable system for the production of therapeutic recombinant proteins. Diverse proteins, such as bacterial and viral antigens, antibodies and, immunotoxins have been successfully expressed in the chloroplast using endogenous and chimeric promoter sequences. In some cases, proteins have accumulated to high level, demonstrating that this technology could compete with current production platforms. This review focuses on the works that have engineered the chloroplast of *C. reinhardtii* with the aim of producing recombinant proteins intended for therapeutical use in humans or animals.

## Introduction

For centuries, algae have been used for human and animal consumption (Spolaore et al. [2006]). More recently algae have been cultivated for the production of fatty acids, secondary metabolites such as pigments, colorants, and bioactive compounds with antitumor, antiviral, and antifungal activities (Borowitzka [1999]; Singh et al. [2005]; Spolaore et al. [2006]). Despite the high number of algae species estimated to populate the planet (Guiry [2012]), only a handful of them are currently used with commercial success. The main species cultivated for human or animal consumption are *Chlorella* and *Spirulina*, while *Dunaliella salina* and *Haematococcus pluvialis* have been cultured for the extraction of β-carotene and astaxanthin, respectively (Borowitzka [1999]).

**Figure 1:**
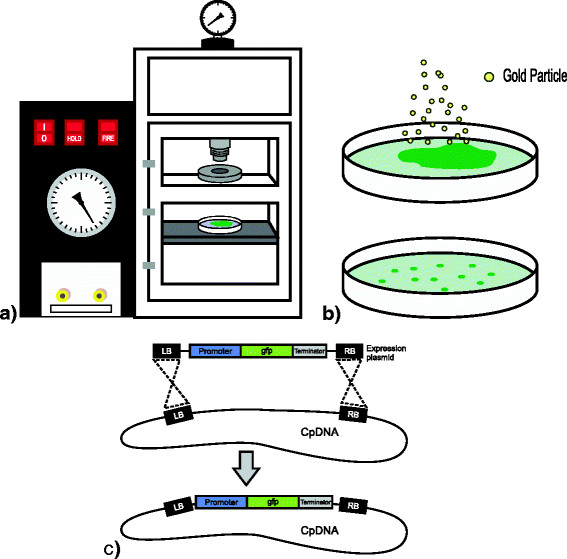
**Chloroplast transformation in** ***Chlamydomonas reinhardtii*. a)** Introduction of foreign DNA material into the chloroplast of *C. reinhardtii* is carried out using a particle bombardment device. The device uses helium gas to accelerate particles towards algae placed at the interior of a vacuum chamber. **b)** Gold or tungsten particles are coated with a plasmid carrying the genes of interest (in this case the green fluorescence protein GFP) and when accelerated penetrate the cells placed on top of selection medium. After a few weeks, transformed cells proliferate in the presence of a selection antibiotic. **c)** When the plasmid carrying the genes reaches de chloroplast, genes integrate into the plastid genome by homologous recombination between regions present in the plasmid (LB and RB) and in the chloroplast genome (CpDNA).

High demand and low productivities are driving the efforts in algal technology towards the development of genetically engineered strains. At the top of the list of the algae species that have been genetically modified comes *Chlamydomonas reinhardtii*, an alga that has been used for a long time as a model for the study of photosynthesis, cell motility, phototaxis, and cell wall biogenesis among other genetic and metabolic processes (Harris [2001]). Transformation protocols exist for the genetic modification of the nucleus (Kindle [1990]), the mitochondria (Remacle et al. [2006]), and the chloroplast (Boynton et al. [1988]). All three compartments have been genetically engineered with different purposes but it has been the chloroplast which has received considerable attention, mainly to demonstrate that the accumulation of recombinant proteins in this organelle is feasible. Chloroplast transformation is carried out using a particle bombardment device as schematised in Figure 1. Chloroplast transformation technology has demonstrated that not only could it compete with current production platforms but it would be better suited for the production of some proteins such as immunotoxins (Tran et al. [2013a]; Tran et al. [2013b]), non glycosilated antigens (Gregory et al. [2012]; Gregory et al. [2013]), and antibodies (Mayfield et al. [2003]; Tran et al. [2009]) which have been difficult to produce in other systems. This review will comment on the most representative cases that have made clear that chloroplast transformation technology to produce therapeutical proteins is beyond the proof-of-concept stage.

## Biopharming in the chloroplast of *C. reinhardtii*

Biopharming refers to the technology that aims, through the use of genetic engineering or, more recently, synthetic biology, to produce health-related products such as antibodies, antigens, human blood components, growth factors, etc. from plants and animals, as opposed to the traditional chemical synthesis in the pharmaceutical industry, the extraction from the natural source or production in bacteria, yeast or mammalian cells (Gimpel et al. [2013]; Specht et al. [2010]). Biopharming in plants and algae has several advantages over bacteria and yeast; three of the most import are: a) a product that is free of human pathogens and more quality-consistent; b) larger quantities can be obtained in a reduced or confined area and; c) low production cost when organisms are grown in economic media.

Microbes, yeast, and mammalian cells have been explored to produce therapeutic proteins (Demain and Vaishnav [2009]), in fact, there are products derived from genetic engineering currently in the market (Leader et al. [2008]). Because *C. reinhardtii* has many advantages over other species, it is not surprising that it has also been proposed as a platform for the production of high-value proteins with therapeutical applications (Specht et al. [2010]). Some of the advantages *C. reinhardtii* has include: a fast growth rate compared to plants and animal cells, it is a Generally Recognized as Safe (GRAS) organism by the Food and Drug Administration from the United States of America, cell cultures can be grown in bioreactors, thus reducing the risk of transgene dispersion, or in open raceways without competing with arable land.

Genetic tools and transformation protocols have been developed for the chloroplast of *C. reinhardtii* for the production of high value molecules for the biotechnological industry (Rosales-Mendoza et al. [2012]; Specht et al. [2010]). However, unlike plants, where chloroplast genetic engineering has been used more widely, for instance, to produce oils, pigments, vitamins, enzymes, or to make drought and salt tolerant plants (Bock [2014]; Maliga and Bock [2011]), chloroplast genetic engineering *C. reinhardtii* has mainly been limited to the production of proteins with therapeutical applications. In Table 1, a comprehensive list of the therapeutic proteins produced in the chloroplast of *C. reinhardtii*, showing the genetic elements used for expression and relevant information can be consulted. Such proteins include: viral and bacterial antigens, antibodies, immunotoxins, and enzymes. In the following section representative cases that have dealt with the production of these proteins will be discussed. For a more complete review of chloroplast genetic tools (Bateman and Purton [2000]; Day and Goldschmidt-Clermont [2011]), synthetic biology in algae (Georgianna and Mayfield [2012]; Gimpel et al. [2013]), and application perspectives (Beer et al. [2009]; Gimpel et al. [2013]) the reader is invited to consult the appropriate references.

**Table 1:** **Overview of recombinant proteins produces in the chloroplast of** ***Chlamydomonas reinhardtii***

**Recombinant therapeutic protein**	**Yield**	**Relevant information**	**Reference**
**VP1-CTB; Protein VP1 from foot and mouth disease virus (FMDV) fused to cholera toxin B (CTB)**	3-4% Total Soluble Protein (TSP)	Demonstrated that the *C. reinhardtii* chloroplast derived VP1-CTB could bind to GM1-ganglioside receptor *in vitro*	(Sun et al. [2003])
**HSV-lsc; Large single chain (lsc) antibody directed against glycoprotein D protein from Herpes simplex virus (HSV)**	Not reported	First report to show that the *C. reinhardtii* chloroplast can efficiently fold antibodies and form disulfide bonds	(Mayfield et al. [2003])
**TRAIL; Tumor necrosis factor-related apoptosis-inducing ligand**	0.43%-0.67% TSP		(Yang et al. [2006])
**M-SAA; Mammary-associated serum amyloid**	3%-5% TSP	M-SAA was shown to generate mucin induction in a human intestinal epithelial cell line. Demonstrated that the *psbA* promoter yields high level or recombinant protein accumulation when the endogenous *psbA* gene is absent	(Manuell et al. [2007])
**CSFV-E2; Classical swine fever virus (CSFV) structural protein E2**	1.5-2% TSP	Subcutaneous immunization of mice with E2 was shown to induced IgG antibodies	(He et al. [2007])
**Human glutamic acid decarboxylase (hGAD65)**	0.25-0.3% TSP	The protein was shown to immunoreact with sera from diabetic mice	(Wang et al. [2008])
**IBDV-VP2; Infectious burial disease virus VP2 protein**	4-0.8% Total cell protein (TCP)	This report looked at the expression of 11 proteins. Nine proteins showed some level of accumulation, while the rest could not be detected. It showed that there are variations in the level of expression even amongst lines obtained with the transformation construct. Authors postulated the existence of the transformosome, a state in which particular genomic characteristics, induced incidentally with transformation, affect, negatively or positively, the expression of the transgene	(Surzycki et al. [2009])
**IHNV-G; Infectious haematopoietic necrosis virus**	< 0.5% TCP		
**IPNV-VP2; Infectious pancreatic necrosis virus**	< 0.3% TCP		
**VP2 protein**	1-0.1% TCP		
**IPNV-VP2 SBC; Infectious pancreatic necrosis virus**	1-0.2% TCP		
**Quorum sensing-regulated gene (LecA) p57**	< 0.5 TCP		
**PCV2; Porcine circovirus type 2**	0.9-0.2% TCP		
**VP-2C**	< 0.5% TCP		
**VP28**	21-0.2% TCP		
**HC-83K7C; Heavy chain human monoclonal antibody against anthrax protective antigen 83 (PA83)**	0.01% dwt	It was shown that the heavy and light chains expressed in trans could assembled into a fully-functional monoclonal antibody against PA83	(Tran et al. [2009])
**LC-83K7C; Light chain human monoclonal antibody against anthrax PA83**			
**CTB-D2; D2 fibronectin-binding domain of** ***Staphylococcus aureus*** **fused to the cholera toxin B subunit**	0.7% TSP	First report to show that an orally-administered alga expressing an antigen in the chloroplast triggers a mucosal and systemic immune response in mice	(Dreesen et al. [2010])
**14FN3; Domain 14 of human fibronectin**	3%-0.15% TSP	This report looked at the expression of seven therapeutic proteins. For three of the proteins, a level of accumulation above 1% was observed, whereas for the rest of the proteins, erythropoietin, interferon β, and proinsulin no protein was detected. Biological activity was evaluated for VEGF and HMGB1	(Rasala et al. [2010])
**VEGF; Human vascular endothelial growth factor**	2%-0.1% TSP		
**HMGB1; High mobility group protein B1**	2.5%-1% TSP		
**acrV2 and vapA2; antigens from the fish pathogen** ***Aeromonas salmonicida***	0.8% and 0.3% TP respectively	Showed that the *psaA* promoter-exon1 element can be used to drive the expression of foreign genes in non-photosynthetic strains	(Michelet et al. [2011])
***Escherichia coli*** **phytase gene (*appA*)**	N.D.	This study showed that algae expressing a bacterial phytase gene in the chloroplast could be lyophilized and administered orally to broiler chicks. The enzyme was active in the gut and reduce the fecal excretion of phytate.	(Yoon et al. [2011])
**Pfs25 and Pfs28; surface proteins from** ***Plasmodium falciparum***	0.5% and 0.2% TSP respectively	First report to show that Pfs25 and Pfs28 can be produced without glycosilation and in a correct conformation recognized by monoclonal antibodies specific to conformational epitopes	(Gregory et al. [2012])
**αCD22PE40; monomeric immunotoxin consisting on the single chain antibody that recognizes the CD22 surface protein from B-cells, fused to domains II and III of exotoxin A (PE40) from** ***Pseudomonas aeruginosa***	0.3%-0.4% TSP	First report to show that immunotoxins can be produced in an eukaryotic system without being toxic to the cell.	(Tran et al. [2013b])
**αCD22HCH23PE40; dimeric version of αCD22PE40**	0.2%-0.3% TSP		
**CtxB-Pfs25;** ***Plasmodium falciparum*** **surface protein 25 fused to the β subunit of the cholera toxin from** ***Vibrio cholera***	0.09% TSP	Demonstrated that the fusion protein can induced IgA antibodies when administered orally as part of a lyophilized powered. However, IgG antibodies could not be elicited with this route of administration	(Gregory et al. [2013])
**αCD22Gel; single chain antibody targeting the CD22 receptor from B-cells, fused to the eukaryotic ribosome inactivating protein, gelonin, from** ***Gelonium multiflorm***	0.2%-0.3% TSP	Demonstrated that immunotoxin can efficiently bind to cancerous B-cells in vitro and kill them without affecting non B-cells	(Tran et al. [2013a])
**αCD22CH23Gel; dimeric version of αCD22Gel**	0.1% - 0.2% TSP		

## Antibodies

Demand for antibodies to treat diverse diseases is constantly increasing and is likely to be difficult to meet in the near future. Antibodies have been difficult to produce in prokaryotic systems and thus have to be produced in eukaryotic systems (Makino et al. [2011]). Most of the commercially available antibodies are currently produced in mammalian cells making the final product expensive and sometimes scarce (Leader et al. [2008]). If antibodies could be efficiently produced in a photosynthetic organism, price would drop and the product would be more accessible to developing countries.

Antibodies have been successfully expressed in the chloroplast of *C. reinhardtii*. Mayfield et al. ([2003]) have expressed a monoclonal antibody directed against a glycoprotein of the herpes simplex virus D (HSV8). The antibody was expressed from a large single chain (lsc) coding sequence, which consisted on the entire IgA heavy chain protein fused to the variable region of the light chain by a flexible linker peptide. The expression of the HSV8-lsc was under the control of the chloroplast *atpA* and *rbcL* promoters. The lsc antibody accumulated as a soluble protein in transplastomic strains (that is, strains with a transgenic chloroplast genome), and was capable of binding to herpes virus protein D *in vitro*. The lsc antibodies formed disulphide bonds and assembled as dimers in the chloroplast.

In another successful case of an antibody produced in *C. reinhardtii* chloroplast, (Tran et al. [2009]) have demonstrated that a monoclonal antibody against a protein from *Bacillus anthracis* could be assembled into a functional tetrameric structure consisting on 2 heavy-chain and 2 light-chain subunits, capable of binding the PA83 antigen from the pathogen *in vitro*. Affinity was determined for the antibodies produced by the alga and an antibody expressed in a mammalian system and were shown to be similar in function. A comparison with antibody 83K7C, expressed in CHO cells, which had been demonstrated to provide protection from anthrax toxicity, in both cell-based assays and animal models, (Tran et al. [2009]) showed that, apart from the lack of glycosylation (inherent to chloroplasts), the chloroplast-produced antibody had the same PA83 binding properties than the antibody produced in the mammalian cell-line. The antibody producing strain was reported to be stable and continued to produce the antibody for more than one year.

## Immunotoxins

Immunotoxins, antibodies linked to a proteic toxin, have found application in cancer treatment as eukaryotic toxins can be coupled to antibodies with cancer-cell specific binding affinity, thus targeting the apoptosis-inducing toxin. Immunotoxins have been difficult to produce in eukaryotic systems, because due to their nature they kill eukaryotic cells, and production in bacteria has had limited success because prokaryotes are unable to fold multiple domain proteins.

The Stephen Mayfield group at the University of California San Diego has shown that immunotoxins can be produced and confined in the chloroplast of *C. reinhardtii*. A single chain antibody recognizing the CD22 surface receptor from B-cells was fused either to domain II and III of Exotoxin A from *Pseudomonas aeruginosa* (Tran et al. [2013b]) or to the ribosome inactivating protein, gelonin, from *Gelonium multiflorm* (Tran et al. [2013a]). In both cases it was demonstrated that the immunotoxins were capable of specifically binding to B-cell *in vitro* and that in the case of the immunotoxin Exotoxin A, survival of mice implanted with a human B-cell tumor, the life-span was extended. The two reports are the first cases in which entire immunotoxins have been produced in eukaryotic cells. This has been possible because the chloroplast served as a confined compartment sequestering the eukaryotic toxin from the cytosol, where it is toxic.

## Antigens for vaccination

Of the total deaths in the world, 25% result from infectious diseases; that number increases to 45% in developing countries (Arntzen et al. [2005]). In humans, vaccination is the most effective mean of inducing resistance to a certain pathogen. The World Health Organization ([WHO]) and the United Nations Children’s Fund ([UNICEF]) have emphasised the need for new production technologies that can satisfy the demand for global immunisation ([UNICEF]; [WHO]). They have also made clear that needle-free, heat-stable, and ease of distribution are important elements for these vaccines to be cost-benefit acceptable.

If antigens intended for vaccination (subunit vaccination) could be produced in GRAS organisms, such as *C. reinhardtii*, the purification process would not be concerned with the removal of toxins or rests of a pathogen, making the process less expensive and the product safer than when it is obtained from microbial cultures. Furthermore, for certain applications, whole GRAS organism (lysed or lyophilized) could even be used as vehicles for administration (Yoon et al. [2011]). A few attempts have been made to express viral or bacterial antigens in the chloroplast of *C. reinhardtii*, for which some examples are given below.

The first subunit viral vaccine produced in the chloroplast of *C. reinhardtii* was the translational fusion of the structural protein VP1, from the Foot and Mouth Disease Virus (FMDV), and the cholera toxin B subunit (CtxB) (Sun et al. [2003]). FMD in livestock can be prevented by subunit vaccination. The structural protein VP1 of FMDV carries critical epitopes, which can induce neutralizing antibodies while the CtxB is a potent mucosal adjuvant that can bind to intestinal epithelial surfaces via GM1 ganglioside receptors. The CtxB-VP1 fusion protein produced in the chloroplast retained both GM1-ganglioside binding affinity and antigenicity. The CtxB-VP1 fusion protein accumulated up to 3% of the total soluble protein (TSP), a 30-fold increase in comparison with the accumulation level obtained for the same antigen when produced from the nuclear genome of alfalfa and *Arabidopsis thaliana* (Carrillo et al. [1998]; Wigdorovitz et al. [1999]).

Bacterial antigens have also been shown to accumulate in the chloroplast of *C. reinhardtii* (Michelet et al. [2011]). Genes *acrV* and *vapA*, from *Aeromonas salmonicida*, an important pathogen causing fish furunculosis were expressed using the *atpA*, *psbA*, and *psbD* promoters, previously reported to produce high levels of expression for various transgenes (Rasala et al. [2011]). The genes were also expressed from a newly proposed promoter, the promoter from gene *psaA* along with exon 1 from the same gene (P*psaA*-exon1). The authors postulated that, since in wild-type strains the *psaA* mRNA is spliced in trans from three separate precursors, the precursor *psaA*-exon1 would over-accumulate in mutants defective in *psaA* transsplicing. The same should happen to a chimeric mRNA with the P*psaA*-exon1. Furthermore, as the unassembled product of *psaA* inhibits the translation of their own mRNA, in a trans-splicing-deficient mutant where *psaA* product cannot be produced, feedback inhibition would be circumvented, and the translation of a recombinant protein expressed under the P*psaA*-exon1 would be higher. It was found that by expressing *acrV* and *VapA* from the P*psaA*-exon1 promoter in a nuclear mutant-deficient on the *psaA* trans-splicing factor, *VapA* accumulated to 0.3% and *AcrV* to 0.8% total protein (TP). In the case of *VapA*, this level was approximately 15 to 30-fold higher than the level of *VapA* present in cells where the nuclear mutation was absent. Even though this increase in the level of accumulation looks attractive, the fact that the transgenic lines obtained are non photosynthetic is a drawback, because, even though lines could be cultivated with acetate as the only carbon source, costs could be reduced if lines were simply grown on CO_2_ and light.

The fact that chloroplasts do not glycosilate proteins might be seen as a drawback. However, this characteristic has been exploited positively for certain proteins, (Gregory et al. [2013]) have successfully expressed the structurally complex non-glycosilated proteins Pfs25 and Pfs28 from *Plasmodium falciparum*, the parasite responsible of malaria. Proteins accumulated to levels of 0.2-0.5% TSP when expressed from the *psbA* promoter in photosynthetic deficient strains. It has been suggested that Pfs25 and Pfs28 could be used to generate antibodies that bind, and neutralize them *in vivo*, thus preventing the formation of *P. falciparum* sporozoites in the mosquitos, which are ultimately responsible for the transmission of the parasite to humans. Producing these proteins in other systems has been difficult as diverse conformations occur and the proteins are glycosylated when produced in eukaryotic systems whilst native Pfs25 and Pfs28 from the parasite are not. When produced in the chloroplast, proteins folded correctly, lacked glycosylation, and generated antibodies that blocked the transmission of the parasite by preventing the formation of the sporozoites in the mosquito *in vitro*. This shows the fitness of the *C. reinhardtii* chloroplast for the expression of properly folded complex proteins.

## Oral delivery of chloroplast-produced antigens

The use of whole lyophilized algae as a vehicle to orally administer biologically active proteins has also been demonstrated, even if only for a limited number of cases. The D2 fribronectin-binding domain from *Staphylococcus aureus* (Georgianna and Mayfield [2012]) and the transmission blocking Pfs25 antigen from *Plasmodium falciparum*, the causative agent of malaria (Gregory et al. [2013]), have been shown to elicit systemic and mucosal responses when fed to mice in a lyophilized powder of algae. The idea that algae expressing antigens could be administered orally seems very attractive because presumably the production of IgG and IgA antibodies could be induced and hence confer protection to the animal or person vaccinated.

In the case of the D2 fribronectin-binding domain from *S. aureus*, specific mucosal (measured by the presence of IgA antibodies) and systemic (measured by the presence of IgG antibodies) immune responses were demonstrated following oral delivery of lyophilized algae expressing the D2 protein. Furthermore, immunized mice were able to reduce pathogen load when *S. aureus* was inoculated in the intestine and the spleen.

In the case of Pfs25 from *P. falciparum*, when it was translationally fused to CtxB and administered orally using a whole-cell lyophilized preparation, IgA antibodies against CtxB and Pfs25 were detected but no IgG antibodies against Pfs25 (Gregory et al. [2013]). This could have been the result of CtxB not being the most appropriate adjuvant for oral vaccination with Pfs25. In contrast, it had been previously demonstrated that when Psf25 was purified and injected into mice an IgG response was observed (Gregory et al. [2012]). If other adjuvants can perform better than CtxB in conjunction with Pfs25 or if some antigen will simply not elicit an immune response when administered orally remains to be investigated.

Even though, these two cases clearly demonstrate that it could be possible to lyophilize algae expressing antigens and use the powder as a vehicle to administer antigens orally, further studies are needed to demonstrate the safety and efficacy of a broader number of antigens. It has been suggested that vaccines produced in edible organisms such as plants, algae or yeast could potentially be administered orally, however, it is not clear yet how dosage could be controlled consistently. Freeze-drying the product could help solve this problem, as the concentration would be easier to determine in a powder (Gregory et al. [2013]). Antigens in lyophilized algae have been shown to be stable at room temperature over a period of months (Dreesen et al. [2010]; Gregory et al. [2013]). A powder that is stable at room temperature may help overcome the problems associated with the cold chain in the distribution of vaccines in developing countries.

## Other therapeutic proteins

In the first report of a recombinant mammalian protein produced in algae, and one of the highest level of protein accumulation yet reported for the chloroplast of *C. reinhardtii*, (Manuell et al. [2007]) have demonstrated that it is possible to produce a bovine mammary-associated serum amyloid (M-SAA) protein at levels above 5% of TSP in the chloroplasts of a photosynthetic strain. M-SAA is found in the colostrum of mammals and can induce mucin synthesis in mammalian gut epithelial cells, which could offer protection against intestinal bacterial and viral infections in new-borns. M-SAA accumulated predominantly as a soluble protein and was shown to be bioactive when assessed by the induction of mucin in a human intestinal epithelial cell line (HT29) *in vitro*.

Manuell et al. ([2007]) have also demonstrated the influence of the product of the *psbA* gene in the expression of genes driven by the *psbA* promoter. When M-SAA was expressed from the *psbA* promoter in a non-photosynthetic strain (that is a line lacking the *psbA* gene) the protein accumulated to 3% TSP. When photosynthesis was restored by introduction of a *psbA* gene under the control of a *psbA* promoter, M-SAA did not accumulate. Interestingly, M-SAA accumulated up to 5% of TSP (12% total protein) when photosynthesis was restored by using the *psbA* gene under the control of the *psbD* promoter. As in both cases the D1 protein was present, there seemed to be a competition between the *psbA* mRNA and the M-SAA mRNA for translation (in which the *psbA* mRNA was favoured). Other reports have also shown that high levels of protein can accumulate from the *psbA* promoter when the endogenous *psbA* gene has been deleted (Rasala et al. [2010]).

The second report for a human protein produced in the chloroplast of *C. reinhardtii* was for Glutamic Acid Decarboxylase 65 (hGAD65), which is a key autoantigen in insulin dependent diabetes mellitus (IDDM) (Wang et al. [2008]). hGAD65 has been identified as an important marker for the prediction and diagnosis of type 1 diabetes, as antibodies against it are present before the onset of the disease and it is believed that tolerance to this autoantigen can be induced using a vaccination-like scheme. Wang et al. ([2008]) transformed the chloroplast of *C. reinhardtii* with the native non codon-optimized hGAD65 gene under control of the chloroplast *rbcL* promoter. The presence of hGAD65 was confirmed by western blotting using anti-GAD antibodies and the level of accumulation accounted for up to 0.3% TSP, a significantly higher value when compared to the 0.04% TSP obtained for a nuclear transgenic plant (Avesani et al. [2010]). The antigenicity of algal-derived hGAD65 was determined by its immunoreactivity with sera from diabetic patients.

In an extensive study, Rasala et al. ([2010]) evaluated the expression of a diverse group of therapeutic human proteins in *C. reinhardtii* chloroplast. Four out of the seven proteins reported in the study accumulated to levels above 1% TSP. All four proteins 10FN3, 14FN3, HMGB1, and VEGF were expressed using the *psbA* and *atpA* promoters and were shown to be biologically active. In a follow up report, (Rasala et al. [2011]) reported the expression of 14FN3, a potential antibody mimic, using a line that lacked the *psbA* gene, and hence, was non-photosynthetic. Lines expressing the 14FN3 from the wild-type *psbA* promoter and from a chimeric promoter (obtained by fusing the 16S rDNA promoter and the *psbA* 5’UTR) were generated. In parallel, using a photosynthetically active strain, the *psbD*, *atpA*, and the chimeric16S rDNA + *atpA* 5′UTR promoters driving the expression of 14FN3 were used to generate transplastomic lines. The wild type *psbA* promoter allowed for the accumulation of 14FN3 to 0.5% TSP but only in the non-photosynthetic strain. The chimeric 16S + *atpA* and 16S + *psbA* promoters also yielded significant amounts of the heterologous protein in photosynthetic strains, 0.23% and 0.21% respectively. Finally, the wild type *atpA* and *psbD* promoters supported only low levels of expression for 14FN3.

Proteins with enzymatic activity intended for animal use have also been produced in the chloroplast of *C. reinhardtii* (Yoon et al. [2011]). Nine versions of a phytase gene of bacterial or fungal origin were used to generate transplastomic lines. Microbial phytases are used as food additives to increase phosphorus utilization from phytate in the livestock industry with the aim of reducing fecal phytates and inorganic phosphate (iP) outputs. (Yoon et al. [2011]) recovered transplastomic algae but only in the case of strain where a full-length, codon-optimized version of the *E. coliappA* gene (E-OPT) phytase was used, enzymatic activity could be detected (10 U/g of algae).

Strains of algae expressing the enzyme phytase were lyophilized and directly added as a food additive, termed Chlasate, to deliver the enzyme with no need of purification. Using this transgenic phytase at 500 U/kg in the diet of young broiler chicks, the fecal phytate excretion was reduced by 43% while the assimilation of iP increased to 41%, as compared to those of chicks fed with only the basal diet. The effectiveness of Chlasate to break down the dietary phytates was comparable with that of a commercial fungal phytase.

To make the final product, cell pastes were directly frozen once with liquid nitrogen, thawed, frozen again and dried with a lyophilizer to obtain a dry cell powder. The one-step frozen-thawing procedure seemed to be sufficient to rupture the *C. reinhardtii* cells and release a functional phytase. The major advantage of using transgenic microalgae for oral delivery is that several processing steps from raw material to the edible final product can be omitted.

## Final remarks and conclusions

It is now clear that chloroplast transformation in *C. reinhardtii* can be consistently accomplished and used to generate transplastomic strains that accumulate recombinant heterologous proteins with therapeutical applications. Although it is still behind the achievements chloroplast transformation has had in land plants has had (Daniell et al. [2009]), the technology has successfully been used in laboratories around the world to produce diverse bacterial, viral, and human proteins.

To achieve high levels of foreign protein accumulation, diverse endogenous and chimeric promoters have been developed. In some cases, recombinant proteins have accumulated up to 20% of the TSP (Surzycki et al. [2009]), although 1-5% TSP is the most common accumulation range for several proteins (Manuell et al. [2007]; Rasala et al. [2010]). In some other cases low to undetectable levels of protein accumulation have been encountered (Rasala et al. [2010]; Surzycki et al. [2009]), stressing the importance of diverse factors in the regulation of gene expression. At present time there is no way of predicting which genetic elements would be better suited for the expression of a particular foreign gene. Some promoters seem to work and yield reasonable levels of expression for some genes whilst for others they seem to be less appropriate (Rasala et al. [2010]).

For some of the proteins revised in this paper, biological activity has been evaluated both *in vitro* and *in vivo*. In the case of antigens intended for vaccination, it has been demonstrated that a systemic and mucosal response can be induced after the administration of a lyophilized powder of the algae expressing the antigen in mice. However, after the purity and efficacy of the antigens has been demonstrated, preclinical trials are needed to demonstrate the safety of the chloroplast derived antigens. Next step would be to carry out clinical trials, though, this may still be a long way from current status; chloroplast-derived vaccines from plants have had a tortuous path from the laboratory to clinical trials (Rybicki [2009]). It is clear that the ultimate goal of producing proteins in the chloroplast of *C. reinhardtii* cannot be considered accomplished until there is an algal produced protein in the market.

Besides being used as bioreactors for the production of recombinant proteins, *C. reinhardtii* and algae in general, have the potential of serving as tools to solve diverse problems. The spectrum of metabolites that could potentially be produced in the chloroplast of *C. reinhardtii* by genetic modification goes from secondary metabolites such as vitamins, carotenoids, and oils to bioplastics and biofuels (such as hydrogen, fatty acids, or solvents), etc. This would alleviate two problems, the ever-increasing worry of a decline in oil production and a reduction in atmospheric CO_2_. Use of algae in bioremediation, removal of toxic compounds from land, water, and air has also been contemplated but poorly studied.

Also, there is a need to expand chloroplast transformation technology to other algae species. Such species should be: 1) easily scalable, with a better ability to grow in open ponds or photobioreactors and, 2) grow faster and have little susceptibility to contamination, but most importantly, 3) strains that depending on the final use are valuable for the production of biopharmaceuticals, secondary metabolites or high energy molecules, such as lipids and sugars. A wide interest in such strains will detonate the development of genetic tools and transformation methods, which has been so far limited to *C. reinhardtii*.

## Competing interests

The authors declare that they have no competing interests.

## Author’s contributions

All authors participated in drafting the manuscript and ALAD and JFU wrote all sections except for the introduction and perspectives, which were written by JABC and ESM. Table 1 was produced by VHPE. All authors have given final approval of the version to be published.
